# Endoscopic treatment of stenosing active tuberculosis to prevent complete bronchial occlusion: a case report and literature review

**DOI:** 10.3389/fmed.2025.1642456

**Published:** 2025-11-12

**Authors:** Kunying Li, Zhenjing Wang, Xia Gao, Taomei Lian

**Affiliations:** 1 Department of Respiratory Endoscopy, Henan Provincial Chest Hospital, Zhengzhou University, Zhengzhou, China; 2 Department of Cardiac Surgery, Henan Provincial Chest Hospital, Zhengzhou University, Zhengzhou, China; 3 Department of Tuberculosis, Henan Provincial Chest Hospital, Zhengzhou University, Zhengzhou, China

**Keywords:** stenosing active tuberculosis, bronchoscopy, needle knife electrocautery, sequential cryotherapy, balloon dilation

## Abstract

**Background:**

Benign central airway scar stenosis, a refractory complication of endobronchial tuberculosis and other inflammatory conditions, often leads to atelectasis, recurrent infections, and respiratory dysfunction. Traditional surgical interventions are associated with significant trauma and high restenosis rates.

**Case presentation:**

This case report describes a 28-year-old female with stenosing active tuberculosis of the left upper lobe bronchus, presenting with cough, high-grade fever, and lobar collapse. The patient underwent transbronchial needle knife electrocautery for precise scar tissue dissection, followed by sequential cryoablation to suppress granulomatous proliferation and balloon dilation for airway remodeling. After five interventional bronchoscopic procedures, complete luminal patency was achieved, with no recurrence observed during 8-month follow-up.

**Conclusion:**

Through this case demonstration and literature review, we highlight the clinical value of combined bronchoscopic techniques (high-frequency electrocautery, cryotherapy, and balloon dilation) in managing stenosing active tuberculosis and preventing complete bronchial occlusion, providing clinicians with a minimally invasive therapeutic alternative.

## Introduction

Benign central airway stenosis refers to non-malignant narrowing lesions of the central airways, including the trachea, main bronchi, and lobar bronchi. Common causes include bronchial tuberculosis, post-intubation scar stenosis, and benign tumors (1). The underlying pathology typically involves airway inflammation and scar tissue proliferation, leading to luminal narrowing or obstruction. With the advancement of interventional pulmonology and bronchoscopy techniques, the diagnostic rate of benign central airway stenosis has increased in recent years. However, treatment remains challenging, particularly in managing complex scar-related stenosis (2). Among all types of benign airway stenosis, scar-related occlusive lesions are the most refractory and difficult to treat. These lesions are typically characterized by dense fibrotic tissue completely obstructing the airway lumen, resulting in severely compromised airway patency. Clinically, patients often present with recurrent infections, cough, wheezing, and may even develop lobar atelectasis or obstructive pneumonia (3). Although conventional surgical resection can relieve the obstruction, it is associated with significant trauma and a high risk of restenosis postoperatively (4). In recent years, bronchoscopic interventional therapy has emerged as a mainstream treatment modality for airway stenosis due to its minimally invasive nature and high repeatability. Among these, the combined application of high-frequency electrocautery, cryoablation, and balloon dilation has shown promising outcomes (5). High-frequency electrocautery enables precise cutting of scar tissue through thermal effects, rapidly reopening occluded airways. Cryotherapy induces cell necrosis through extreme cold, thereby inhibiting granulation tissue proliferation and reducing intraoperative bleeding. Balloon dilation mechanically remodels the airway structure by exerting radial force. Studies have demonstrated that this combined approach yields an immediate efficacy rate of up to 67.8% in treating scar-related stenosis (6) Currently, there are relatively few reported cases of bronchoscopic combination therapy for stenosing active tuberculosis, making it difficult to draw definitive conclusions regarding its therapeutic efficacy and prognosis. This case report presents the diagnosis and treatment process of a patient with stenosing active tuberculosis of the left upper lobe bronchus. By integrating a review of the relevant literature, the aim is to analyze bronchoscopic interventional treatment strategies and provide evidence to inform and optimize clinical management approaches.

## Case presentation

**Figure 1:**
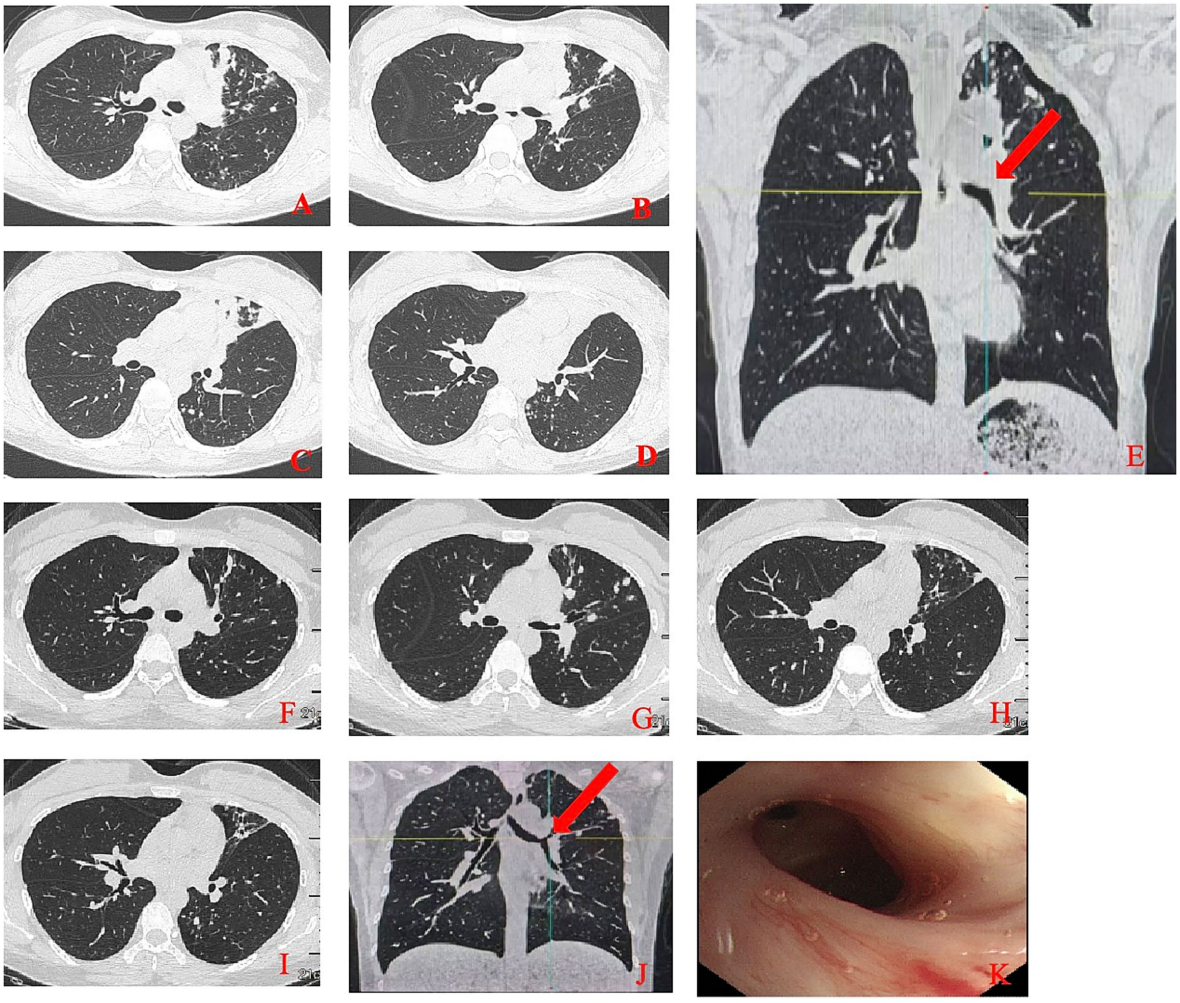
Initial and follow-up chest CT Imaging with corresponding bronchoscopic findings. **(A–E)** Initial chest CT scan showed complete occlusion (arrow, **E**) of the left upper lobe bronchus and multiple infectious lesions in the left lung. **(E–J)** Follow-up chest CT showed significant resolution of the lesion (arrow, **J**) with a patent left upper lobe bronchus. **(K)** The left upper lobe bronchus with smooth mucosa and stable scar tissue.

A 28-year-old female presented to our hospital on July 5, 2024, with a chief complaint of intermittent cough for 6 months, worsening over the past 20 days, and high fever for 1 day. She reported the onset of a dry, non-productive cough without any apparent trigger 6 months prior, with no associated symptoms such as fever, chest tightness, chest pain, or hemoptysis. She had sought medical attention at a local clinic 4 months earlier, where she received oral antibiotics and antitussive medications, resulting in slight symptom relief. Approximately 20 days before admission, her cough worsened, accompanied by low-grade afternoon fevers. A chest CT performed at a local hospital revealed multiple patchy opacities in the left lung. Bronchoscopic examination showed severe cicatricial stenosis at the orifice of the left upper lobe bronchus. Next-generation sequencing (NGS) of the bronchoalveolar lavage fluid was positive for *Mycobacterium tuberculosis*. Meanwhile, genetic mutation testing for first-line drug resistance revealed resistance to isoniazid. A diagnosis of pulmonary tuberculosis was made. One day before admission, the patient developed a sudden high fever, with a peak temperature of 40 °C, prompting her to seek emergency care at our facility, where she was admitted with a diagnosis of pulmonary tuberculosis. Physical examination revealed absent breath sounds in the left upper lung field, while breath sounds in the remaining lung fields were clear, with no audible dry or moist rales. The patient was alert and oriented, in good general condition, with normal appetite, sleep, bowel and bladder function, and no significant change in body weight. A chest CT scan performed after admission (Figures 1A–E) showed complete occlusion of the left upper lobe bronchus and multiple infectious lesions in the left lung. Bronchoscopic interventional therapy was planned as the proposed treatment approach.

**Figure 2:**
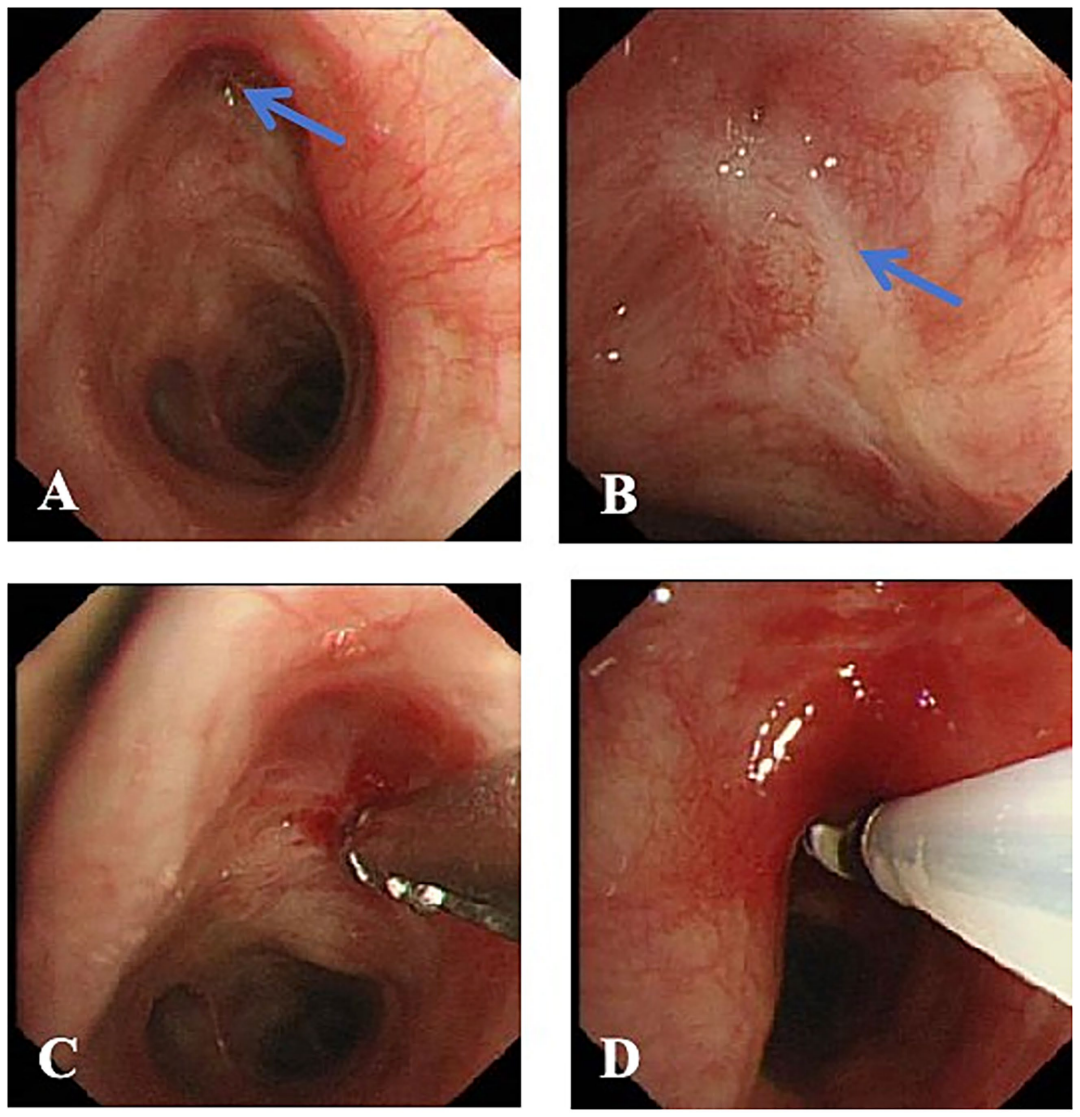
Bronchoscopic image from the first intervention after admission. **(A)** Orifices of the left upper (blue arrow) and lower lobe bronchi. **(B)** Scar occlusion (blue arrow) at the orifice of the left upper lobe bronchus. **(C)** Exploration with biopsy forceps attempting to remove scar tissue, confirming complete scar occlusion at the orifice. **(D)** Cryotherapy applied to the scar-occluded orifice of the left upper lobe bronchus.

**Figure 3:**
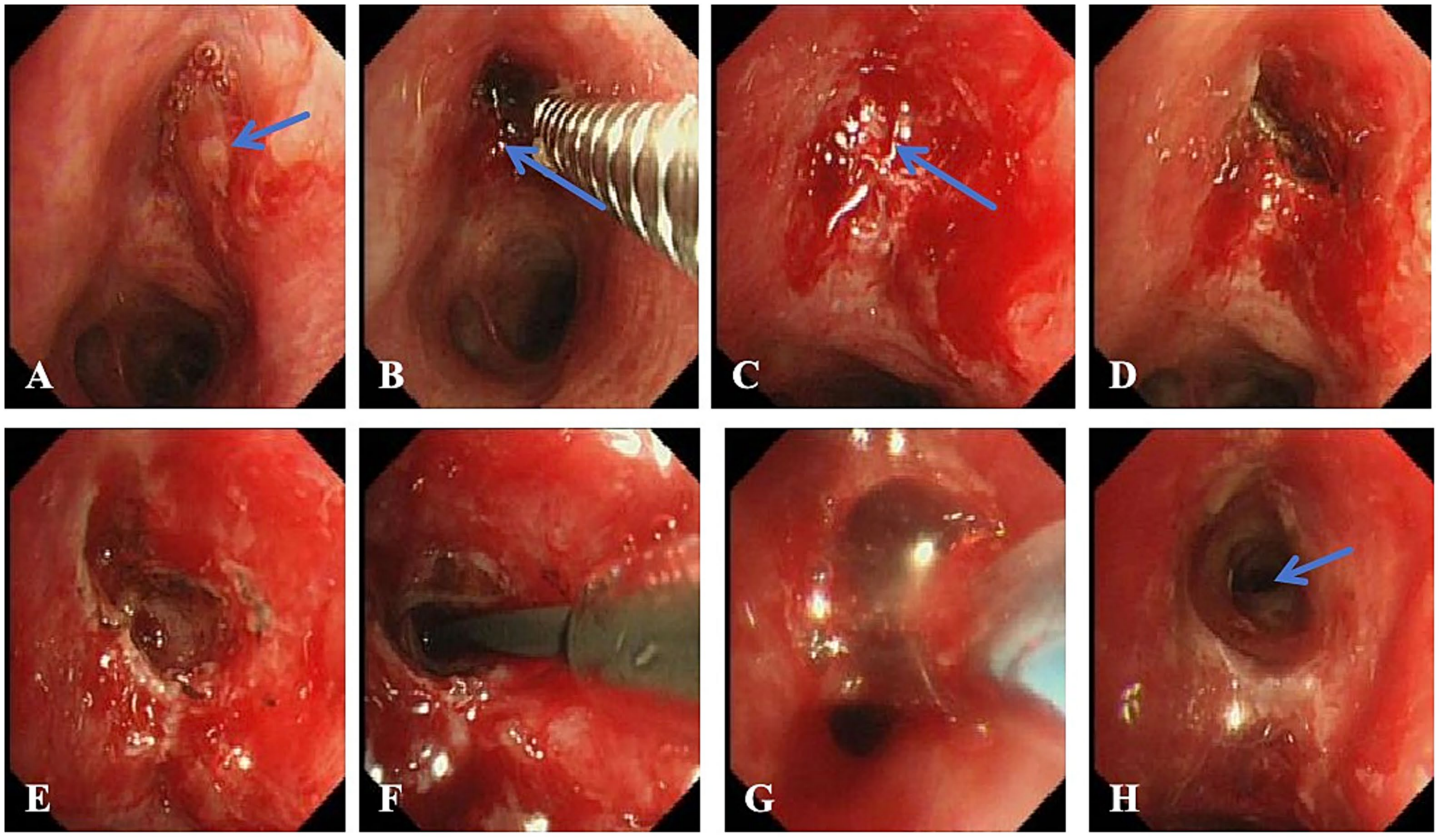
Bronchoscopic images of the second interventional procedure. **(A)** Inflammatory granulation tissue (blue arrow) observed at the orifice of the left upper lobe bronchus. **(B)** Inflammatory tissue (blue arrow) removed using biopsy forceps. **(C)** After removal, the bronchial orifice remained occluded (blue arrow), indicating that cryotherapy alone was insufficient. D/E. Needle-type electrocautery used to incise and release scar tissue, followed by repeated probing with biopsy forceps and guidewire to access the distal airway. F. Balloon dilation catheter advanced into the stenotic segment of the left upper lobe bronchus under guidewire guidance. **(G)** Balloon dilation performed. **(H)** The lumen of the left upper lobe bronchus was re-exposed (blue arrow), with a small amount of purulent secretion observed.

After admission, the anti-tuberculosis regimen was initiated with rifampin injection (R, 0.45 g once daily), ethambutol hydrochloride tablets (E, 0.75 g once daily), oral pyrazinamide tablets (Z, 0.75 g once daily), and levofloxacin injection (LFX, 0.5 g once daily), continuing for 6 months. In addition, the patient underwent multiple sessions of bronchoscopic interventional therapy, as detailed below: First Intervention (July 15, 2024): Bronchoscopic examination revealed complete cicatricial occlusion of the left upper lobe bronchial orifice (Figure 2). Exploration with biopsy forceps was unsuccessful due to the tight occlusion. Cryotherapy was performed to loosen the fibrotic tissue, an ERBE cryotherapy equipment and soft flexible cryoprobe with a diameter of 1.9 mm and a probe measuring 10 mm were used (Erbe Elektromedizin GmbH, Tuebingen, Germany), with liquid CO_2_ as the freezing source. The multi-point freezing method was adopted, 30–120 s for each part. Second Interventional Procedure (1 Week Post-Procedure): Bronchoscopy still showed occlusion of the left upper lobe bronchus, accompanied by mild inflammatory granulation tissue proliferation (Figure 3A). The proliferative tissue was removed using biopsy forceps (Figure 3B). After removal, the bronchial orifice remained occluded (Figure 3C). A needle-type electrocautery probe was then used for scar incision and release (Figures 3D,E), followed by sequential cryotherapy. Successful passage of the biopsy forceps and guidewire into the distal airway was achieved. Balloon dilation was subsequently performed using a three-stage airway balloon catheter (outer diameters of 8, 9, and 10 mm) provided by Boston Scientific (Figure 3F). Balloon inflation pressures ranged from 304 to 811 kPa, gradually increased in steps. Each dilation was maintained for 3 min (Figure 3G). Post-procedure, the lumen of the left upper lobe bronchus was visibly reopened (Figure 3H), although local mucosa appeared congested, edematous, and eroded. Following successful endoscopic restoration of the left upper lobe bronchial lumen, approximately 10 mL of whitish purulent secretions were aspirated and submitted for microbiological analysis. Microscopic examination showed a negative acid-fast bacilli smear using the Ziehl-Neelsen concentration method. Molecular testing by Xpert MTB/RIF assay detected *Mycobacterium tuberculosis* complex DNA and demonstrated the absence of rifampin resistance, as no rpoB gene mutation was detected. Microbiological culture revealed mycobacterial growth after 26 days of incubation, with subsequent antigen detection confirming the identity as *M. tuberculosis* complex. Drug susceptibility testing indicated that the *Mycobacterium tuberculosis* isolate was resistant to isoniazid and streptomycin but remained susceptible to rifampicin, ethambutol, amikacin, para-aminosalicylic acid, levofloxacin, moxifloxacin, prothionamide, and capreomycin.

**Figure 4:**
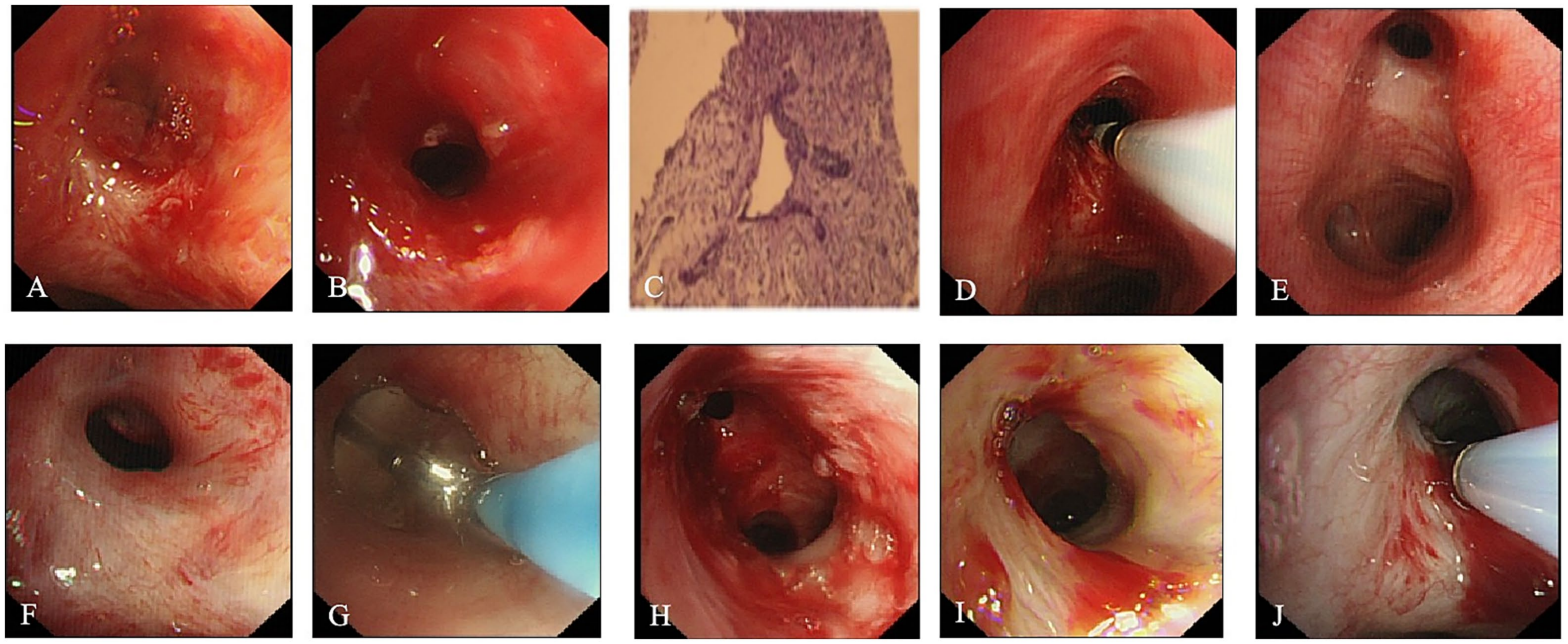
Bronchoscopic images from the third to fifth interventional treatments. **(A)** Inflammatory tissue hyperplasia (blue arrow) at the orifice of the left upper lobe bronchus, obstructing most of the lumen. **(B)** Removal of inflammatory tissue (blue arrow) using biopsy forceps, with specimens sent for pathological analysis. **(C)** Inflammatory tissue biopsy specimen obtained during the third intervention. Pathological results confirming inflammatory lung tissue. **(D)** Cryotherapy during the third intervention. **(E–H)** Bronchoscopic images from the fourth intervention (performed 10 days after the third session). **(E)** Openings of the left upper and lower lobe bronchi. **(F)** Stenosis is observed at the opening of the left upper lobe bronchus, and balloon dilation therapy was performed again **(G)**. Post-dilation outcome **(H)**. **(I,J)** Bronchoscopic images from the fifth intervention (performed 20 days after the fourth session). **(I)** Shows an enlarged opening of the left upper lobe bronchus compared to previous findings. **(J)** The left upper lobe bronchial orifice (underwent cryotherapy, with stable scar formation).

One week later (third intervention): Bronchoscopy revealed inflammatory tissue proliferation at the orifice of the left upper lobe bronchus, obstructing most of the lumen (Figure 4A). Biopsy forceps were used to remove the lesion tissue, and pathology confirmed inflammatory lung tissue (Figures 4B,C). Following tissue removal, cryotherapy was performed (Figure 4D), and budesonide nebulization was added to the clinical treatment.

Ten days later (fourth intervention): Bronchoscopy showed that although there was scar stenosis at the left upper lobe bronchus orifice, the airway was more patent than before, and the scar appeared stable (Figures 4E,F). Balloon dilation was performed again using Boston Scientific airway triple balloon catheters with diameters of 8, 9, and 10 mm. The balloon pressure ranged from 304 to 811 kPa, gradually increasing from low to high, and dilation was maintained for 3 min (Figure 4G). Post-dilation, the bronchial lumen was noticeably enlarged (Figure 4H), followed by local cryotherapy.

Twenty days later (fifth intervention): Bronchoscopy showed a patent left upper lobe bronchus orifice with reduced stenosis compared to before; the mucosa was smooth with localized congestion, and the scar tissue of the bronchial wall appeared stable (Figure 4I). Local cryotherapy was administered again (Figure 4J).

After 8 months of treatment, chest CT reexamination (Figures 1F–J) showed significant resolution of the lesions and a patent left upper lobe bronchus. Bronchoscopic examination revealed a clear airway in the left upper lobe bronchus with smooth mucosa and stable scar tissue (Figure 1K). Although mild stenosis remained, it no longer impaired ventilation function, indicating a favorable long-term treatment outcome. During follow-up, the patient reported no significant cough or dyspnea, was able to carry out normal daily activities and work, and showed no signs of symptom recurrence, with a marked improvement in quality of life.

## Discussion

This report presents a case of benign central airway scar stenosis caused by stenosing active tuberculosis of the left upper lobe bronchus in a young female patient. The patient’s condition was effectively managed through a combination of transbronchial needle knife electrocautery, sequential cryotherapy, and balloon dilation performed via bronchoscopy. This multimodal bronchoscopic approach allowed precise scar dissection, suppression of granulomatous tissue proliferation, and airway remodeling, resulting in restoration of airway patency after five interventions. The patient remained symptom-free with no restenosis during an 8-month follow-up period. A comprehensive literature review further supports the efficacy and safety of this combined minimally invasive treatment for stenosing active tuberculosis. These findings underscore the potential of bronchoscopic interventional techniques as valuable alternatives to traditional surgery, offering less trauma and better long-term outcomes for patients with complex benign airway stenosis.

To systematically evaluate the application of bronchoscopic combined interventional therapy in bronchial cicatricial occlusion, we conducted a literature search in PubMed, CNKI, and the Chinese Medical Journal Full-text Database using the keywords “occlusion” AND “bronchoscopy” AND “tuberculosis.” A total of 1,364 articles published between 1954 and 2025 were identified. After excluding studies involving non-cicatricial occlusion, non-interventional treatments, and non-English/Chinese publications, eight articles were included that specifically addressed bronchoscopic interventional treatment for tuberculous bronchial cicatricial occlusion. These articles collectively reported on eight patients. To further assess the efficacy of bronchoscopic treatment for tuberculous cicatricial occlusion, we summarized the clinical characteristics, treatment protocols, and follow-up outcomes of nine confirmed cases of tuberculous cicatricial bronchial occlusion (including the present case), as detailed in Table 1.

**Table 1:** Summary of reported cases of tuberculous bronchial cicatricial occlusion treated with bronchoscopic interventional therapy.

Author	Year	Gender	Age (years)	Symptoms	Occlusion site	Treatment	Number of treatments	Complication	Relapse-free time (months)
Qiusheng Jing (7)	2022	Female	32	Cough, expectoration	Left main bronchus	Bronchoscopic YAG laser, cryotherapy, balloon dilation, silicone stent placement	4	-	4
Yukari Ichikawa (8)	2023	Female	77	Respiratory failure	Right main bronchus	Bronchoscopic balloon dilation	3	-	21
Yoshio Nakano (9)	2024	Female	20	Dyspnea	Right main bronchus	Transbronchial biopsy, virtual bronchoscopy-guided balloon dilation	2	-	-
Haosu Zhou (10)	2025	Female	49	Chest tightness, dyspnea	Right main bronchus	ECMO-assisted laser therapy, high-frequency electrocautery, and balloon dilation via bronchoscopy	Multiple sessions	-	-
Yongping Gao (11)	2022	Female	30	Dyspnea	Left main bronchus	Bronchoscopic needle-knife incision, balloon dilation, CO₂ cryotherapy	Multiple sessions	Pneumothorax	11
Xin Zhang (12)	2022	Female	42	Cough, chest tightness, dyspnea	Right main bronchus	Bronchoscopic high-frequency electrosurgical recanalization	5	Tracheal mediastinal perforation	44
Li Luo (14)	2023	Female	30	Cough, expectoration, dyspnea	Left main bronchus	Bronchoscopic high-frequency electrosurgical knife, cryotherapy, biopsy forceps for scar removal, mechanical and balloon dilation	7	-	7
Jieru Lin (15)	2023	Female	26	Breathing, phonation difficulties	Subglottic upper trachea	Bronchoscopic laser ablation, balloon dilation, cryotherapy, and T-tube placement	Multiple sessions	-	3
Present case	2025	Female	28	Cough, fever	Left main bronchus	Bronchoscopic needle-knife electrocautery, sequential cryotherapy, balloon dilation	5	-	8

The average age of the nine patients was 37.1 years (range: 20–77 years), and all were female. A study by Rongjuan Zhuang et al. (7) indicated that central airway stenosis caused by tuberculosis predominantly occurs in young women. Among the patients, 4 (44.44%) presented with cough, 3 (33.33%) with dyspnea, and another 3 (33.33%) with shortness of breath. Other symptoms included fever and productive cough. The sites of bronchial occlusion were evenly distributed between the left and right main bronchi (44.44% each). An analysis of the current case along with eight other reported cases of tuberculous bronchial cicatricial occlusion suggests that bronchoscopic combined interventional therapy offers significant clinical advantages. The therapeutic approach utilized in the present study included needle-type electrocautery, cryotherapy, and balloon dilation. After five treatment sessions, the patient achieved full airway patency, with no recurrence observed during an eight-month follow-up period. Similarly, the literature demonstrates that combination interventional therapy patients experienced symptom relief, increased bronchial lumen diameter, and eventual airway stabilization. The complication rate was low, with only two cases (22.22%) reporting procedure-related events such as tracheomediastinal perforation or pneumothorax, indicating a favorable safety profile (8, 9). Compared to conventional surgical methods, bronchoscopic interventional therapy is less invasive, facilitates faster recovery, and is associated with a lower complication rate, thus enhancing postoperative outcomes (10). The follow-up results of the nine cases reviewed (up to 44 months) revealed no recurrence, suggesting that bronchoscopic combination therapy may be effective in maintaining long-term airway patency. This potential advantage may be attributed to the multimodal mechanism of action: precise scar tissue incision via needle-type electrocautery, inhibition of granulation tissue formation through cryotherapy, and structural airway remodeling achieved with balloon dilation. The synergistic application of these techniques may significantly reduce the risk of restenosis.

Bronchial cicatricial occlusion caused by tuberculosis presents unique challenges. Its pathological features are characterized by chronic inflammation accompanied by fibroblast proliferation and collagen deposition. Compared with other causes of bronchial stenosis, tuberculous scarring tends to be denser and more fibrotic, often containing substantial necrotic tissue, thereby making treatment more difficult (11). Currently, common bronchoscopic interventional techniques for managing bronchial scarring include laser ablation, high-frequency electrosurgical incision, cryotherapy, balloon dilation, and stent placement. The choice of technique should be guided by the type and stage of the stenosis. Balloon dilation involves advancing a deflated balloon catheter into the stenotic segment under guidewire assistance. The balloon is then inflated using high-pressure saline infusion, applying mechanical radial force to induce longitudinal micro-tears in the airway wall. This triggers a subsequent fibrotic remodeling process that helps maintain lumen patency. Balloon dilation is particularly effective in cases with a shorter disease duration (<3 months), a stenotic segment length ≤1 cm, and a luminal obstruction rate <70%. However, in patients with extensive fibrosis or complete airway occlusion, multiple sessions are often required to achieve satisfactory outcomes (12). Laser ablation facilitates the precise removal of hyperplastic scar tissue and rapid restoration of airway patency by converting photonic energy into thermal energy to induce localized thermal effects. This technique is associated with minimal intraoperative bleeding, a clear visual field, and a low incidence of complications. However, its use is limited in cases of extrinsic airway compression, active bleeding, or severe infection (13). Cryotherapy is considered particularly effective for tissues with relatively high water content. It employs cryogenic materials and devices to generate extremely low temperatures, causing rapid intracellular ice crystal formation that disrupts cell membranes, halts local blood flow, and induces chronic pathological changes such as microthrombosis, ultimately leading to tissue necrosis. Cryotherapy causes minimal injury to tracheobronchial tissues, allows for mucosal regeneration, and is associated with few complications. It is suitable for treating obstructive tracheobronchial lesions, including benign and malignant tumors, foreign bodies, blood clots, and necrotic tissue within the airway. However, it is not ideal for emergency airway decompression (14). Endotracheal stent placement is a rapid and effective method for relieving central airway stenosis. Nevertheless, it carries a risk of complications such as granulation tissue proliferation and recurrent infections, which may result in restenosis or occlusion (15). In clinical practice, specific combined treatment protocols are often dynamically adjusted based on bronchoscopic findings. For mixed-type stenosis accompanied by cartilaginous destruction, stent implantation can provide necessary mechanical support; however, the risk of granulation tissue formation must be carefully weighed. For instance, in the case reported by Qiusheng Jing (16), a silicone stent was temporarily placed to mitigate this risk. In cases of subglottic stenosis (17), the use of a T-tube in combination with localized cryotherapy may offer both airway stabilization and effective secretion management. Notably, bronchoscopic interventions under extracorporeal membrane oxygenation (ECMO) support, as described in the case by Haosu Zhou (18), have opened new therapeutic windows for critically ill patients. However, such approaches require close multidisciplinary collaboration to minimize procedural risks. In addition, the introduction of novel technologies has expanded the therapeutic options for tuberculous bronchial scar occlusion. For example, L-shaped silicone stents have demonstrated efficacy in preventing stent migration and alleviating airway narrowing or obstruction (19), while self-expanding metallic stents have shown promise in reducing the risk of restenosis (13). Although these emerging techniques have shown potential in preliminary studies, their effectiveness in the management of tuberculous bronchial scar occlusion requires further investigation.

This case report details the successful management of post-tuberculous left main bronchial stenosis using advanced bronchoscopic techniques. However, it is critical to underscore that the success of any interventional procedure is contingent upon the effective control of the active tuberculous infection. Timely diagnosis and standard anti-TB chemotherapy remain the cornerstone of management, aiming to prevent such severe complications from the outset. For the complex stenosis that developed in this patient, we demonstrated the efficacy of a combination of balloon dilation and cryotherapy. It is also important to acknowledge that surgical alternatives (e.g., lobectomy, bronchoplasty) remain a crucial definitive option for cases with extensive disease not amenable to bronchoscopic interventions. The future direction should involve a multidisciplinary approach that tailors the treatment pathway, from endoscopy to surgery, based on individualized anatomical and functional assessments.

In conclusion, this study highlights the clinical value of combined bronchoscopic techniques (high-frequency electrocautery, cryotherapy, and balloon dilation) in managing stenosing active tuberculosis and preventing complete bronchial occlusion, providing clinicians with a minimally invasive therapeutic alternative. Future research should focus on larger, prospective studies to further validate the safety, efficacy, and long-term outcomes of combined bronchoscopic interventions for tuberculous bronchial stenosis.

## Data Availability

The original contributions presented in the study are included in the article/supplementary material, further inquiries can be directed to the corresponding author.
